# Identification of Silkworm Hemocyte Subsets and Analysis of Their Response to Baculovirus Infection Based on Single-Cell RNA Sequencing

**DOI:** 10.3389/fimmu.2021.645359

**Published:** 2021-04-30

**Authors:** Min Feng, Junming Xia, Shigang Fei, Ruoxuan Peng, Xiong Wang, Yaohong Zhou, Pengwei Wang, Luc Swevers, Jingchen Sun

**Affiliations:** ^1^ Guangdong Provincial Key Laboratory of Agro-Animal Genomics and Molecular Breeding, College of Animal Science, South China Agricultural University, Guangzhou, China; ^2^ Insect Molecular Genetics and Biotechnology, National Centre for Scientific Research Demokritos, Institute of Biosciences and Applications, Athens, Greece

**Keywords:** scRNA-seq, Lepidopteron, Hemocytes, BmNPV, *Bombyx mori*

## Abstract

A wide range of hemocyte types exist in insects but a full definition of the different subclasses is not yet established. The current knowledge of the classification of silkworm hemocytes mainly comes from morphology rather than specific markers, so our understanding of the detailed classification, hemocyte lineage and functions of silkworm hemocytes is very incomplete. *Bombyx mori* nucleopolyhedrovirus (BmNPV) is a representative member of the baculoviruses and a major pathogen that specifically infects silkworms (*Bombyx mori*) and causes serious losses in sericulture industry. Here, we performed single-cell RNA sequencing (scRNA-seq) of hemocytes in BmNPV and mock-infected larvae to comprehensively identify silkworm hemocyte subsets and determined specific molecular and cellular characteristics in each hemocyte subset before and after viral infectmadion. A total of 20 cell clusters and their potential marker genes were identified in silkworm hemocytes. All of the hemocyte clusters were infected by BmNPV at 3 days after inoculation. Interestingly, BmNPV infection can cause great changes in the distribution of hemocyte types. The cells appearing in the infection group mainly belong to prohemocytes (PR), while plasmatocytes (PL) and granulocytes (GR) are very much reduced. Furthermore, we found that BmNPV infection suppresses the RNA interference (RNAi) and immune response in the major hemocyte types. In summary, our results revealed the diversity of silkworm hemocytes and provided a rich resource of gene expression profiles for a systems-level understanding of their functions in the uninfected condition and as a response to BmNPV.

## Introduction

Insects have an efficient and potent innate immune system to discriminate and respond to invading pathogenic microorganism (1). The innate immune system of insects is divided into humoral defenses that include the production of soluble effector molecules (like antimicrobial peptides) and cellular defenses like phagocytosis and encapsulation that are mediated by hemocytes (2). A wide range of hemocytes exists in insects but a full definition of the different subclasses is not yet established (1). In Lepidopteran insects such as the silkworm, hemocytes are generally classified into five major subsets including prohemocytes (PR), plasmatocytes (PL), granulocytes (GR), spherulocytes (SP), and oenocytoids (OE) based on morphology and function (2–4). Generally, prohemocytes are considered as multipotent progenitor cells or stem cells giving rise to the other subsets (5). Plasmatocytes and granulocytes are the only hemocyte subsets capable of adhering to foreign surfaces, and together usually comprise more than 50% of the hemocytes in circulation in the larval stages (3). Furthermore, plasmatocytes and granulocytes are involved in most cellular defense responses (2, 3). Oenocytoids are in rich in prophenoloxidase and mainly participate in melanization, whereas the functions of spherulocytes are unknown (6, 7).

Because our current knowledge of the classification of silkworm hemocytes mainly comes from morphology and cytochemistry rather than from specific genetic markers, our detailed understanding of hemocyte lineages and functions is very limited. Importantly, if hemocyte subsets cannot be distinguished well, the molecular responses of each hemocyte subset during the invasion of exogenous pathogenic microorganisms remain inaccessible. In addition, although some research teams have studied the hematopoietic lineage of the silkworm (5, 6), still little is known about silkworm hemocyte lineage trajectories starting from precursor cells or the existence of putative intermediate states during the differentiation process towards mature cell types. Hence, it is important and interesting to thoroughly characterize the molecular signatures and responses of all the precursor and mature hemocyte subtypes during homeostasis and after exposure to pathogenic microorganisms.

Baculoviruses are a very diverse group of invertebrate-specific DNA viruses with genome sizes varying from about 80 to over 180 kbp, that encode between 90 and 180 genes, which bring harm to economically important insects, but also have been harnessed for biotechnological applications such as insect pest control and the expression of heterologous proteins (8). *Bombyx mori* nucleopolyhedrovirus (BmNPV), a representative member of baculoviruses, is a major pathogen that specifically infects silkworms (*B. mori*) and causes serious losses in the sericulture industry (9). However, no effective measures are currently available to prevent BmNPV infection. With respect to sericulture, many silkworm strains have very little resistance to BmNPV infection which can lead to widespread mortality and population collapse. The general high virulence of baculovirus infections has also been exploited for their successful application as biocontrol agents to kill pests. However, although methods such as bulk RNA-seq and proteomics have been used in recent years to try to clarify the interaction between baculovirus and the host (9–14), major gaps remain regarding the host response to the virus, especially at the level of cellular immunity. Hence, the elucidation of the response of different hemocyte subsets to BmNPV infection is a powerful approach to explore the insect cellular immune response to baculovirus infection.

Single-cell RNA sequencing (scRNA-seq) has already been applied to investigate the immune response under conditions of virus infection (15, 16). In particular, scRNA-seq technology is widely used to characterize the response of various cell types to severe acute respiratory syndrome corona virus 2 (SARS-CoV-2) infection (17–19). In *Drosophila*, one of the major model organisms, the cell lineage characteristics in many tissues have been identified using scRNA-seq, such as the midgut (20), blood cells or hemocytes (21–23), ovary (24, 25), and brain (26). However, the application of scRNA-seq technology to determine cell subsets and cell lineages is rare in other insects. Moreover, to our knowledge, scRNA-seq technology has not been applied yet to study the responses of specific insect cell (sub)types to viral infections.

Here, we have performed scRNA-seq on silkworm hemocytes in BmNPV-infected larvae and uninfected larvae to comprehensively identify hemocyte subsets and characterize their specific molecular and cellular responses after viral infection.

## Materials and Methods

### Silkworm and BmNPV Infection

Larvae of silkworm (*B. mori*, Dazao strain) were reared with fresh mulberry (*Morus* sp.) leaves and reared under constant environmental conditions of 28°C and 60-70% humidity. Recombinant BmNPV-EGFP (Enhanced Green Fluorescent Protein), as a reporter virus, was constructed by the Bac-to-Bac Baculovirus Expression System and kept in Guangdong Provincial Key Laboratory of Agro-animal Genomics and Molecular Breeding. Newly molted fifth-instar silkworm larvae were injected with either 10 μL of BmNPV-EGFP (10^5.8^ TCID_50_/0.1 mL) or phosphate-buffered saline (PBS) (injection control). Blue Light Gel Imager (Sangon Biotech, China) was used to monitor the spread of green fluorescence in infected larvae on a daily basis until whole body expression was achieved.

### Preparation of Single Hemocytes in Suspension

At three days after BmNPV infection, 3 mL of hemolymph was collected from pools of twenty BmNPV-infected animals or twenty controls on ice and centrifuged at 500 g for 2 min at 4°C. The hemocyte pellet was washed twice using 1 mL of cold Grace’s insect medium (Gibco, USA) supplemented with 10% FBS (Gibco, USA), which was followed by filtering through a 40 µm cell strainer (Biosharp, China) and centrifugation at 500 g for 2 min at 4°C. The hemocyte pellet was resuspended using 200 µL PBS (calcium and magnesium-free, Gibco, USA) supplemented with 0.04% bovine serum albumin (BSA) (Solarbio, China). Cell viability and number were assessed by 0.4% trypan blue staining and cell counting using a hemocytometer. High quality hemocyte preparations were subjected to single cell encapsulation by 10X Genomics v3 kit (10x Genomics, USA).

### Single Hemocyte Encapsulation and Sequencing

Single cell encapsulation, complementary DNA (cDNA) library synthesis, RNA-sequencing and data analysis were completed by Gene Denovo (Guangzhou, China). The single-cell suspensions were bar-coded and reverse-transcribed into scRNA-seq libraries using the Chromium Single Cell 3’ Gel Bead-in Emulsion (GEM), Library and Gel Bead Kit (10x Genomics, USA) according to the manufacturer’s protocol. Briefly, single silkworm hemocytes were barcode-labeled and mixed with reverse transcriptase into GEMs, then the cDNA library was amplified using PCR with the sequencing primers R1 and R2, and subsequently ligated to Illumina sequencing adapters with P5 and P7 flow cell binding sites. Finally, the cDNA libraries were sequenced on the Illumina 10x Genomics Chromium platform (Illumina Novaseq 6000).

### ScRNA-Seq Data Processing and Analysis

The latest published version of the silkworm genome (SilkDB3.0) was used in the present study (27). Since SilkDB3.0 does not contain the mitochondrial genome, we have added silkworm mitochondrial genome data (NCBI Reference Sequence: NC_002355.1) in the subsequent analysis. The raw scRNA-seq data were aligned, filtered, and normalized using Cell Ranger (10x Genomics) software (Cell Ranger 3.1.0). A read is considered as exonic if at least 50% of it intersects an exon, intronic if it is non-exonic and intersects an intron, and intergenic otherwise. A read that is compatible with the exons of an annotated transcript, and aligned to the same strand, is considered mapped to the transcriptome. If the read is compatible with a single gene annotation, it is considered uniquely (confidently) mapped to the transcriptome. Only reads that are confidently mapped to the transcriptome are used for Unique Molecular Identifier (UMI) counting.

Seurat is a popular R package that can perform quality control (QC), analysis, and exploration of scRNA-seq data, which was originally developed as a clustering tool for scRNA-seq data (28). In this study, the cell subset was grouped by graph-based clustering based on the gene expression profile of each cell in Seurat. After removing of low-quality cells, data were normalized. Normalized expression levels were used to perform canonical correspondence analysis (CCA) to correct for the batch effect which was followed by data integration to carry out Z-score normalization. The normalized data were clustered using principal component analysis (PCA) and visualized with t-distributed Stochastic Neighbor Embedding (t-SNE) (29) or Uniform Manifold Approximation and Projection (UMAP) (30). Other data analyses including standardization, difference of gene expression, and marker gene screening were also achieved by Seurat.

Notably, scRNAseq data were validated for analysis according to the following QC criteria: 1) 200 < gene counts < 4000 per cell; 2) UMI counts < 30, 000 per cell; 3) percentage of mitochondrial genes < 25%. After normalizing the data using Seurat, gene expression level in scRNA-seq was calculated as log [1 + (UMI A ÷ UMI Total) × 10000]. It should be noted that when performing gene quantification, ‘UMI Total’ does not include ‘UMI BmNPV’.

### Differentially Expressed Genes (DEGs) Analysis Per Cluster

Likelihood-ratio test (31), performed on single cluster cells against all other cells, was used to identify differentially expressed genes (DEGs) in single clusters based on differential expression. Up-regulated DEGs in each cluster were identified by the following criteria: 1) p value ≤ 0.01; 2) log2FC ≥ 0.360674 (log2FC means log fold change of the average expression between the two groups); and 3) percentage of cells in a specific cluster where the gene is detected > 25%. Gene Ontology (GO) and Kyoto Encyclopedia of Genes and Genomes (KEGG) pathway enrichment analysis were further carried out based on gene expression levels to identify the main features of each cluster. The top 5 genes in each cluster were selected as the potential marker genes according to the result of DEGs analysis. Then the expression distribution of each marker gene among clusters was visualized by bubble diagrams.

### Viral Genes Analysis in Cell Clusters of the BmNPV-Infected Group

To determine the state of BmNPV infection in each hemocyte cluster, the expression of viral genes and viral load were analyzed in the different cell clusters in the BmNPV-infected group. Viral gene expression was analyzed using Cell Ranger, based on the BmNPV genome (GenBank: JQ991008.1). The ‘viral load’ of a cell in scRNA-seq analysis was based on the number of UMIs that map to the BmNPV genome and expressed as a percentage of total UMI content of a given cell (16). In the present study, infected hemocytes were divided into three categories of viral-load states: low (< 5%), medium (between 5% and 20%), and high (> 20%).

### DEGs Analysis in Cell Clusters Between BmNPV-Infected and Uninfected Groups

To explore the response of each hemocyte cluster to BmNPV, we further analyzed the DEGs between the BmNPV-infected group and the control group using Seurat software under the condition of a minimum of 3 cells per cluster. New identities were set up as “group_cluster” or “group_cell type” for analysis (32). A hurdle model in MAST (Model-based Analysis of Single-cell Transcriptomics) (33) was used to find DEGs for a group in one cluster. DEGs between the BmNPV-infected and control groups were identified by the following criteria: 1) |log2FC| ≥0.25; 2) p value_adj ≤ 0.05; and 3) percentage of cells where the gene is detected in a specific cluster > 25%. Identified DEGs were subsequently subjected to GO and KEGG pathway enrichment analysis.

### Analysis of Bulk RNA-Seq Data

Bulk RNA-seq raw data of silkworm plasmatocytes (PL) and granulocytes (GR) were kindly provided by Dr Kui Zhang and Prof Hongjuan Cui (unpublished data, Southwest University, China). Plasmatocytes used in their study were obtained by culturing hematopoietic organs (from *B. mori*, Dazao strain) *in vitro* and granulocytes were obtained by culturing the adherent cells collected from the Dazao silkworm from which the hematopoietic organs were removed (Kui Zhang’s doctoral thesis, in Chinese, 2017). Since the flow cytometry sorting method cannot be used to separate silkworm hemocytes due to the lack of known marker genes and corresponding interacting specific antibodies, the PL and GR isolates described above are therefore considered to be impure preparations.

After removing low-quality reads and ribosomal RNA, high quality clean reads were mapped to the latest version of the silkworm genome (SilkDB 3.0) using HISAT2. 2.4 (34). The mapped reads were assembled using StringTie v1.3.1 (35). FPKM (fragment per kilobase of transcript per million mapped reads) values were calculated to quantify genes’ expression abundance and variations, using StringTie software (35). Pearson’s correlation analysis was used to investigate the relationships between bulk RNA-seq data of PL, GR and hemocyte clusters in scRNA-seq based on levels of gene expression.

### Hemocytes Classification and Count

Hemocytes were prepared from BmNPV-infected (wild-type BmNPV BV) and uninfected silkworms as described above and stained with the acridine orange/propidium iodide double staining kit (BioRab, China) before evaluation. After classification according to cell morphology by fluorescence and light microscopy (4, 36), the proportion of the different hemocyte cell types (PR, GR, PL, OE, SP) was determined (3 independent samples).

### Pseudo Temporal Ordering of Cells

Single cell trajectory was analyzed using a matrix of cells and gene expression levels by Monocle 2 (Version2.6.4) (37). Monocle reduces the space in which the cells were embedded to two dimensions and orders the cells (parameters used: sigma= 0.001, lambda = NULL, param.gamma = 10, tol= 0.001). The cells in the BmNPV-infected group were defined as the starting point of the pseudotime analysis. Once the cells were ordered, the trajectory (with tree-like structure, including tips and branches) could be visualized in the reduced dimensional space.

## Results

### Single-Cell Transcriptomics Identifies 20 Distinct Cell Clusters in the Hemolymph Collected From BmNPV-Infected and Control Silkworms

**Figure 1:**
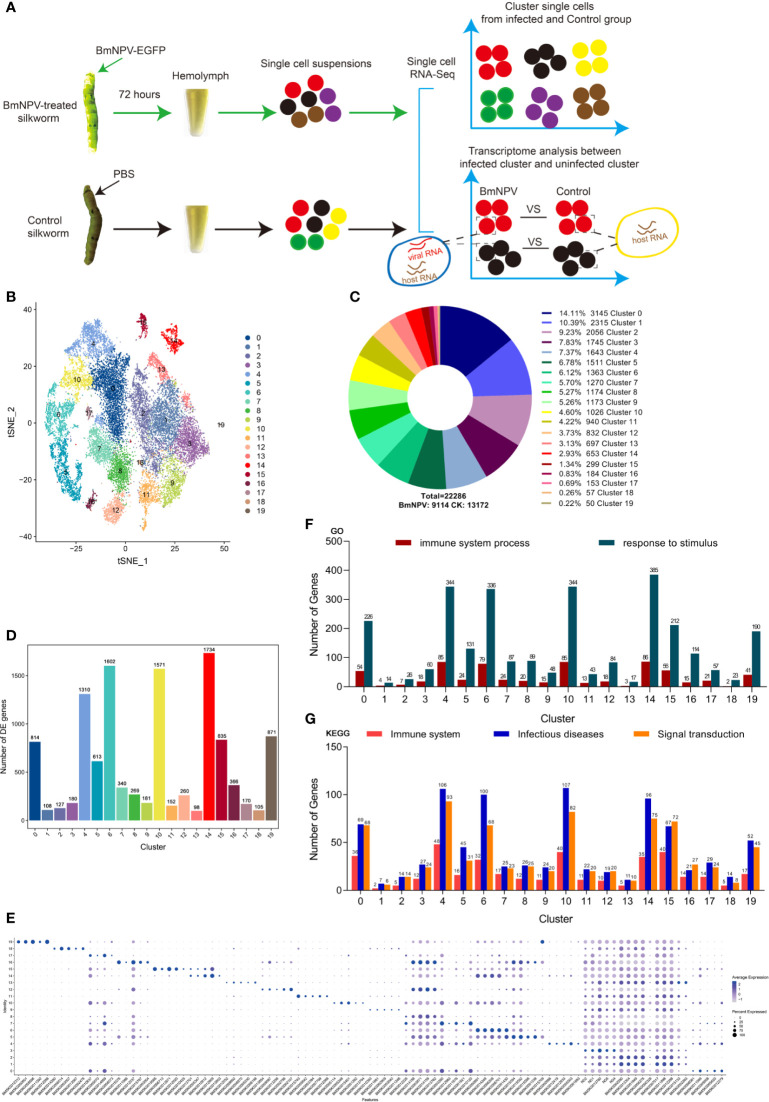
Single-cell profiling of cell populations in silkworm hemolymph. **(A)** Schematic illustration of the experimental workflow. Twenty larvae for each group were treated with BmNPV or PBS and sacrificed at 72 hours post treatment. Hemolymph of the twenty larvae in each group were pooled as one sample for single-cell sequencing (scRNA-seq) analysis. In each single cell, mRNAs of host and virus were simultaneously measured, allowing comparison of the transcriptome profile in each cell cluster under the infection or control condition. **(B)** t-Distributed Stochastic Neighbor Embedding (t-SNE) projection representing the 20 clusters of cells identified in the silkworm hemocyte pools (unified set of control and BmNPV infection samples). **(C)** Number of cells in each cluster and their proportional distribution in the total hemocyte dataset. **(D)** Number of up-regulated differentially expressed genes(DEGs)in each cluster. **(F)** The statistics of genes involved in “immune system process” and “response to stimulus” GO terms as analyzed in each cluster. **(G)** The statistics of genes involved in “Immune system”, “Infectious diseases” and “Signal transduction” KEGG pathways as analyzed in each cluster. **(E)** Top 5 DEGs (x axis) identified in each cluster (y axis). Dot size represents the fraction of cells in the cluster that express the gene; intensity indicates the mean expression (Z-score) in expressing cells, relative to other clusters.

We used the 10x Genomics platforms to perform 3′ scRNA-seq on pooled hemocytes collected from BmNPV-infected silkworm and PBS-treated controls (**Figure 1A**). Details on the statistics of scRNA-seq are summarized in **File S1**. A total of 22,286 cells (BmNPV-infected: 9,114; Control: 13,172) were profiled and 20 distinct clusters were obtained that can be visualized using t-SNE (**Figures 1B, C**). From cluster 0 (3145 cells, 14.11%) to cluster 19 (50 cells, 0.22%), the number of cells gradually decreases (**Figure 1C**). However, no known marker genes exist that can be used to specify different types of silkworm hemocytes, in contrast to the robust classification of hemocyte subgroups in *Drosophila* (21, 22). Next, we identified the up-regulated DEGs of each cluster and analyzed the enrichment of specific groups of genes involved in distinct cellular processes. Silkworm hemocyte clusters were detected with a large number of up-regulated DEGs, especially cluster 14 (1734 DEGs), 6 (1602), 10 (1571), 4 (1310), 19 (871), 15 (835), 0 (814) and 5 (613) (**Figure 1D** and **File S2**). The expression levels and the percentage of cells expressing the top five genes in each cluster are shown in a dot plot (**Figure 1E** and **File S3**). These genes need to be confirmed in future research and are proposed to be used as marker genes for each cluster of silkworm hemocytes (**Figure 1E**). Through GO and KEGG analysis on all up-regulated DEGs in each cluster, we found that genes involved in immune-related host response processes are mainly enriched in cluster 0, 4, 6, 10, 14, 15 and 19 (**Figures 1F, G**). We speculate that these hemocyte clusters are the main effectors in the silkworm hemolymph that respond to external stimuli. However, the clusters with the highest numbers of DEGs and enrichment in immune-related host response processes correspond to the clusters that are predominant in control hemocytes (clusters 0, 4, 6, 10, 14, 15 and 19) (see further below).

To check that the strategy of combining the hemocytes of the control and BmNPV groups into one set has no effect on the result outcome, the hemocytes of the separate groups of control and BmNPV infection were mapped to the unified set (BmNPV+CK) using cell-specific barcodes. In addition, the hypervariable genes that were identified for cell cluster classification in scRNA-seq were compared between individually analyzed groups and the unified set (BmNPV+CK). **Figure S1** shows that the agreement between the two clustering approaches is relatively high (**Figures S1A, S1B**), especially for the cells in the control group (**Figure S1B**). Furthermore, irrespective of whether control and BmNPV infection groups were analyzed separately or as a unified set, most of the hypervariable genes to cluster the hemocytes were the same (**Figure S1C**). These results confirm that the analysis of the unified set of cells from both experimental groups (as used in the remainder of this study) represents a valid approach.

### Immune-Related Gene Expression Signatures of Each Cluster

**Figure 2:**
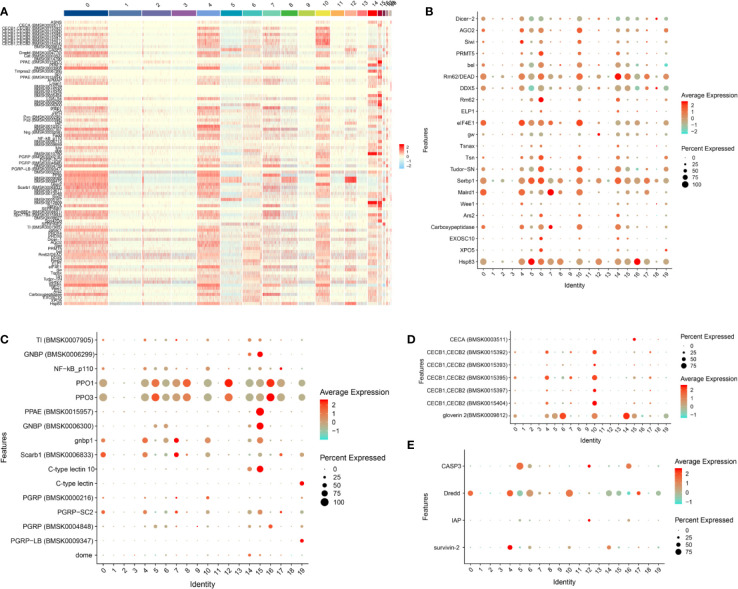
Landscape of expression of RNAi and immune-related genes in silkworm hemocyte clusters. **(A)** Heatmap showing the normalized expression (Z-score) of all immune-related DEGs in various cells of hemocyte clusters. **(B–E)** Dot plot representing DEGs belong to “RNAi” **(B)**, “innate immune pathway” **(C)**, “antimicrobial peptide genes” **(D)** and “cell death” **(E)** categories in hemocyte clusters based on average expression. Color gradient of the dot represents the expression level, while the size represents the percentage of cells expressing any gene per cluster.

According to the previously reported silkworm immune-related gene data (including RNAi-related genes) (38, 39), a total of 93 immune-related genes were screened from cluster 0 to 19 and their expression level was presented as a heat map (**Figure 2A** and **File S4**). We can observe that most of these immune-related genes were highly expressed in clusters 0, 4, 6, 7, 10, 14 and 15 (**Figure 2A**). It is well established that RNAi is a major antiviral defense mechanism in insects (40). Besides RNAi, many other innate immune pathways have been proposed to be involved in antiviral defense such as the Imd and Toll pathways, the JAK/STAT pathway, autophagy and apoptosis (40–42). The dot plot showed that most of the RNAi-related genes were highly expressed in clusters 0, 4, 5, 6, 10, 14, 15, 16 and 19 (**Figure 2B**). However, a relatively low number of innate immune pathway-related genes are differentially expressed in each subgroup (**Figure 2C**). Notably, the antimicrobial peptide (AMP) gene *Cecropin A* was only detected in cluster 15 and *gloverin 2* was highly expressed in several clusters such as cluster 6, 10 and 14 (**Figure 2D**). AMP genes of the *Cecropin B* family are highly expressed in hemocyte cluster 4 and especially cluster 10 (**Figure 2D**). Caspase-8 (Dredd), a molecular switch for apoptosis, necroptosis and pyroptosis (43), was found to be highly expressed in most of the cells of several clusters such as clusters 0, 4, 6 and 10 (**Figure 2E**). *Caspase-3* encodes a key enzyme playing an essential role in both exogenous and endogenous apoptotic pathways (44). We found that the silkworm *Caspase-3* is only highly expressed in cluster 5 and 16, while expression was restricted to a small number of cells in cluster 12 (**Figure 2E**). Death pathways are major defense mechanisms against baculovirus infection (45) and these data therefore indicate that hemocyte clusters 0, 4, 5, 10, 12, 16 and 17 could use this strategy, although different death pathways may be engaged in different clusters.

In conclusion, hemocyte clusters 0, 4, 5, 6, 7, 10, 14, 15, 16 and 19 may be involved in the antiviral responses after BmNPV infection.

### Major Impact of BmNPV Infection on Hemocyte Identities and Physiologies

**Figure 3:**
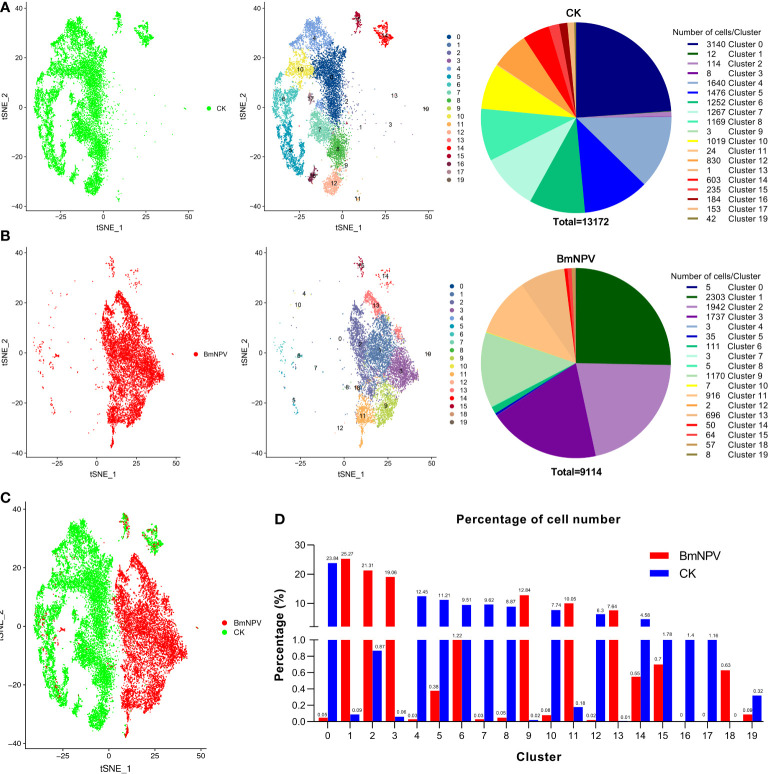
Hemocyte heterogeneity between BmNPV-infected and mock-infected larvae. **(A, B)** t-SNE displaying all identified cell types and their cell number in PBS-treated (CK) **(A)** and BmNPV-infected larvae **(B)**. **(C)** t-SNE displaying the high heterogeneity in hemocyte clusters between the BmNPV-infected and control groups. **(D)** The distribution of cells in each cluster between the BmNPV-infected and control groups.

We further compared the distribution of each silkworm hemocyte cluster in the BmNPV-infected and the control groups. In the uninfected sample, we detected all previously described cell types from cluster 0 to cluster 19, except cluster 18 (**Figure 3A**), although cell numbers in clusters 1, 2, 3, 9, 11 and 13 were much reduced. Interestingly, hemocyte clusters which may be involved in the antiviral response such as clusters 0, 4, 5, 6, 7, 10, 14, 15, 16 and 19 are the main components of uninfected silkworm hemocytes (**Figure 3A**). On the other hand, only a few cells were detected in these hemocyte clusters (0, 4, 5, 7, 8, 10, 12, and 19) in the BmNPV-infected sample (**Figure 3B**). Clusters 16 and 17 were absent in the BmNPV-infected sample, while only two cells were detected in cluster 12 (**Figure 3B**). Correspondingly, few cells of hemocyte clusters 1, 3, 9, 11, and 13 were detected in the control, while a large number of cells were detected in these clusters in the infection group (**Figures 3A, B**). Comparing the cellular landscapes, we therefore observe that the cells in the BmNPV-infected group and the control group were for a large part distributed in different clusters (**Figure 3C**). After analyzing the proportion of control and infected cells in each cluster, it was found that hemocyte clusters which may be involved in the antiviral response (0, 4, 5, 6, 7, 10, 14, 15, 16 and 19) showed a striking depletion in the BmNPV-infected sample (**Figure 3D**). On the other hand, clusters 1, 2, 3, 9, 11, 13, and 18 are the main components of the silkworm hemocytes in the BmNPV-infected group (**Figure 3D**).

### High Viral Load Is a Characteristic of Major Infected Cell Types

**Figure 4:**
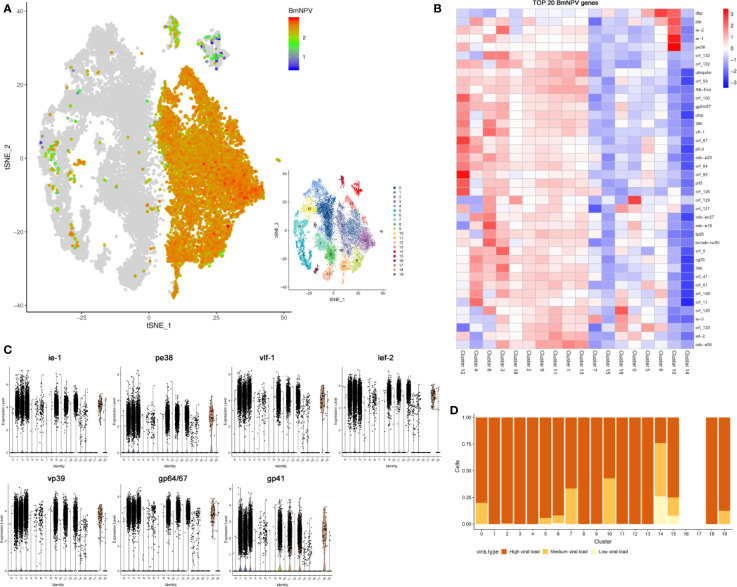
Analysis of viral gene expression and viral load in BmNPV-infected hemocytes. **(A)** t-SNE displaying the normalized expression (Z-score) of all viral genes in each BmNPV-infected cell. **(B)** Heatmaps showing the normalized expression (Z-score) of the top 20 of highly expressed BmNPV genes in each cell cluster of the BmNPV-infected group. **(C)** Expression levels of viral early genes (*ie-1* and *pe38*), late genes (*vlf-1* and *lef-2*) and characteristic viral genes (*vp39*, *gp64* and *gp41*) in hemocyte clusters. **(D)** The proportion of cells with different viral load in each cluster. Shown are the percentages of low (light yellow), medium (yellow), and high (brown) viral-load states (y axis) within the population of infected cells in each cluster (x axis).

**Figure 5:**
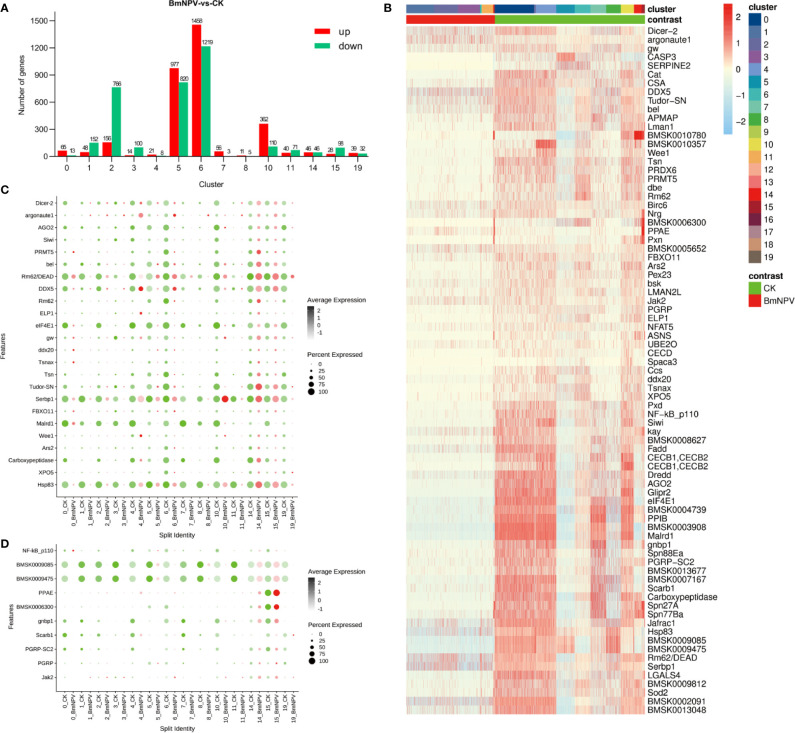
DEGs analysis between BmNPV-infected and control cells within cell clusters. Seurat was used for DEGs analysis for clusters with more than 3 cells. **(A)** Histogram showing all of up-regulated (red) and down-regulated DEGs (green) in BmNPV-infected cells compared to control cells within selected clusters. **(B)** Heatmap showing the normalized expression (Z-score) of the RNAi and immune-related DEGs in various cells of hemocyte clusters: cells from the BmNPV-infected sample are compared with the control sample. **(C, D)** Dot plot representing DEGs belonging to “RNAi” **(C)** and “innate immune pathway” **(D)** categories in hemocyte clusters: cells from the BmNPV-infected sample are compared with the control sample. The black gradient represents the expression level, while the size of the dots represents the percentage of cells expressing any gene per cluster. Red dots represent the BmNPV-infected group and green dots represent controls.

Since the viral mRNA is poly-adenylated, scRNA-seq can capture both viral and host mRNAs within each individual cell (**Figure 1A**). Individual BmNPV-infected cells could therefore be identified following alignment of viral genes with the BmNPV genome. Unexpectedly, viral genes can be detected in every cell of the BmNPV-infected sample (**File S5**), illustrating widespread and efficient infection of hemocytes by baculovirus in the silkworm. In **Figure 4A**, the abundance of viral gene expression in individual cells is illustrated (values of the expression abundance of each viral gene are presented in **File S5**). In most infected cells, high expression of viral genes is observed (**Figure 5A**). The top 20 of highly expressed BmNPV genes in each cell cluster are illustrated in **Figure 4B** and presented in detail in **File S5**. The violin plots show that viral early genes (*ie-1* and *pe38*) as well as late genes (*vlf-1* and *lef-2*) and more specifically *vp39* (encoding the major capsid protein), *gp64/67* (envelope gene) and *gp41*(an essential gene required for the egress of nucleocapsids from the nucleus) (8) are all highly expressed in most cells of each hemocyte cluster in the BmNPV-infected sample at 3 dpi (**Figure 4C**). When focusing on the distribution of the viral loads (defined as the proportion of viral UMIs in the total UMI content of a given cell) within infected cells of different hemocyte clusters, it was observed that infected cells in most of the clusters (1, 2, 3, 4, 8, 9, 11, 12, 13 and 18) carried a high viral load of at least 20% (**Figure 4D**). Additionally, infected cells within cluster 0, 5, 6, 7, 10, 15 and 19 displayed variability of viral-load, although the vast majority still maintained high viral-load states, except for those in cluster 14 (**Figure 4D**). Together, these data suggested that all hemocytes could be infected by BmNPV and that the majority of infected cells carried a high viral-load at 3 dpi.

### DEGs Analysis Between BmNPV-Infected and Control Cells Within Cell Clusters

To characterize the host responses against to BmNPV infection, we calculated the differential expression of genes between cell populations of the BmNPV-treated and control samples using Seurat (applied on clusters with more than 3 cells). Compared to the control sample, it was found that most of the host genes were up-regulated after BmNPV infection in hemocyte clusters 0, 5, 6, 7, and 10 (**Figure 5A** and **File S6** and **Figure S2**). In fact, many up-regulated genes belong to the ribosomal pathway (**File S6**). Evaluation of the landscape of immune/RNAi-related DEGs shows that their expression was mainly inhibited in hemocyte clusters during BmNPV infection (**Figure 5B**). Specifically, DE RNAi-related genes, including the genes encoding Dicer-2 and Argonaute-2, the core factors of the siRNA pathway, are mainly down-regulated in the BmNPV-infected sample (**Figure 5C**). It is worth noting that several DE RNAi-related genes are highly expressed in clusters 14 and 15 after BmNPV infection (**Figure 5C**). Additionally, the DEGs involved in pathogen pattern recognition and innate immune pathways are also inhibited in the BmNPV-infected sample (**Figure 5D**). Thus, we speculate that on the third day after infection with BmNPV, the host’s antiviral system in hemocytes is in a suppressed state.

### Identification of Specific Cell Types in the Hemolymph of Silkworm

Since there are no recognized marker genes for various types of silkworm hemocytes, it was not obvious to classify the identified clusters 0-19 into the specific subgroups described in the literature such as plasmatocytes, granulocytes, spherulocytes and oenocytoids (3). However, we tried to distinguish clusters 0-19 based on the genes that are highly expressed in each subgroup of silkworm hemocytes determined by morphology in the literature. These potential marker genes found in the literature (7, 46, 47) were subjected to the BLAST function of SilkDB 3.0 (27) to obtain the new gene IDs of the database. Specifically, Nakahara et al. (7) determined that *serine protease homolog 1*(BMSK0013968)*, βGRP3* (BMSK0006299) and *paralytic peptide* (BMSK0007609) were expressed only in plasmatocytes and no other hemocyte subset; that t*SCR-C* (BMSK0015652), *CatB* (BMSK0010120), *HP1* (BMSK0003908) and *PGRP* (BMSK0004739) were detected in granulocytes; that *PPBP1* (BMSK0014159) and *PPBP2* (BMSK0013765) were strongly expressed in oenocytoids; and that *cathepsin L-like protein* (BMSK0005696) can be used as a spherulocyte-specific molecular marker in the silkworm. Moreover, it was reported that *integrinaPS3* (BMSK0006871) could be used as a specific marker for silkworm granulocytes (46). Additionally, in Kui Zhang’s doctoral thesis (In Chinese, 2017), it was found that *Integrin β3* (BMSK0001792) and *BmSCRB8* (BMSK0013677) were strongly expressed in plasmatocytes and granulocytes, respectively. Moreover, the promoter of *Integrin β3* can drive EGFP specifically in plasmatocytes but not in other hemocyte types (47).

**Figure 6:**
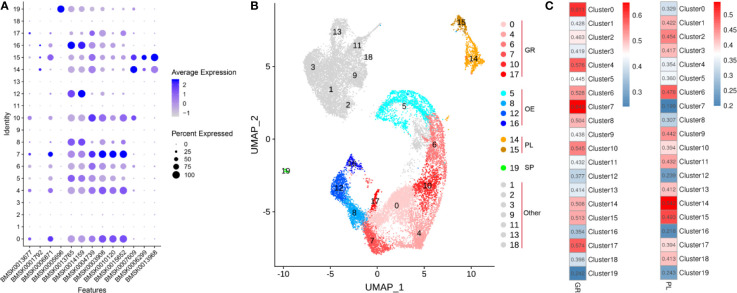
Specific cell types in the hemolymph of silkworm larvae. **(A)** Potential marker genes collected from published literature and their expression in hemocyte clusters 0 to 19. Gene identities of SilkDB3.0 correspond to the following genes in literature: BMSK0013677 (*BmSCRB8*), BMSK0001792 (*Integrin β3*), BMSK0006871 (*integrinaPS3*), BMSK0005696 (*cathepsin L-like protein*), BMSK0013765 (*PPBP2*), BMSK0014159 (*PPBP1*), BMSK0004739 (*PGRP*), BMSK0003908 (*HP1*), BMSK0010120 (*CatB*), BMSK0015652 (*SCR-C*), BMSK0007609 (*paralytic peptide*), BMSK0006299 (*βGRP3*), BMSK0013968 (*serine protease homolog 1*). The color gradient of the dot represents the expression level and the size reflects the percentage of cells expressing these genes per cluster. **(B)** UMAP displaying the plasmatocytes (PL), granulocytes (GR), spherulocytes (SP), oenocytoids (OE) and unclassified cells (Other-mainly in BmNPV-infected group). **(C)** Pearson’s Correlation analysis between bulk-RNAseq data from partially purified GR and PL and scRNA-seq data of different cell clusters based on gene expression levels.

We explored the expression of the genes mentioned in literature in clusters 0-19 and displayed them in bubble plot (**Figure 6A**). Based on these potential marker genes, we first identified cluster 19 as spherulocytes based on *cathepsin L-like protein* (BMSK0005696) expression (**Figures 6A, B**). Next, we assigned clusters 14 and 15 as plasmatocytes because of the presence of DEGs *serine protease homolog 1*(BMSK0013968), *paralytic peptide* (BMSK0007609), *βGRP3* (BMSK0006299) and *Integrin β3* (BMSK0001792) (**Figures 6A, B**). We further assigned cluster 0, 4, 6, 7, 10 and 17 as granulocytes because the presence of markers such as *SCR-C* (BMSK0015652), *CatB* (BMSK0010120), *HP1*(BMSK0003908), *PGRP* (BMSK0004739), *BmSCRB8* (BMSK0013677) and *integrinaPS3* (BMSK0006871) (**Figures 6A, B**). Finally, clusters 5, 8, 12 and 16 were identified as oenocytoids because of high expression of *PPBP1* (BMSK0014159) and *PPBP2* (BMSK0013765) (**Figures 6A, B**). Moreover, the Pearson’s Correlation analysis between bulk RNA-seq of partially purified granulocytes and plasmatocytes and scRNA-seq showed that clusters 0, 4, 6, 7, 10 and 17 have high correlation with granulocytes and that clusters 14 and 15 likely correspond with plasmatocytes (**Figure 6C**). This result increases the reliability of the assignment of scRNAseq clusters as plasmatocytes and granulocytes in this study.

**Figure 7:**
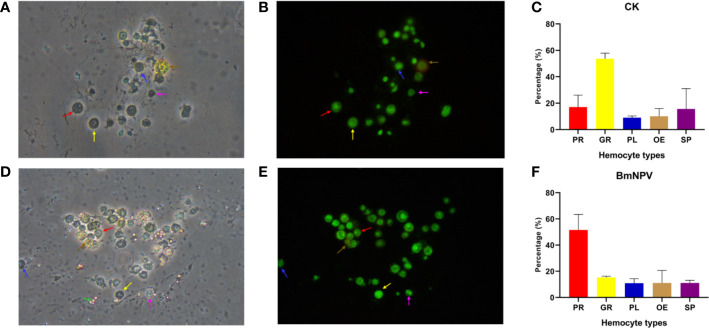
Hemocytes classification and quantification. Newly molted fifth-instar silkworm larvae (Dazao strain) were injected with either 10 μL of wild BmNPV BV (10^5.8^ TCID_50_/0.1 mL) or phosphate-buffered saline (PBS) (injection control). Three days later, hemocytes were collected and stained with acridine orange and propidium iodide. **(A, B)** Morphology of normal silkworm hemocytes under bright field **(A)** and green fluorescence (488nm) **(B)**. **(C)** Statistics of the proportion of hemocyte subgroups in normal silkworms. **(D, E)** Morphology of hemocytes of BmNPV BV-infected silkworm under bright field **(D)** and green fluorescence (488nm) **(E)**. **(F)** Statistics of the proportion of hemocyte subgroups in BmNPV-infected silkworms. The red arrow, yellow arrow, blue arrow, brown arrow and purple arrow point to prohemocytes (PR), granulocytes (GR), plasmatocytes (PL), oenocytoids (OE) and spherulocytes (SP), respectively. Assays were performed three times. In particular, in the infection group, the BmNPV polyhedron pointed by the green arrow in the bright field **(D)** is not visible by green fluorescence **(E)**.

Granulocytes, plasmatocytes, spherulocytes, and oenocytoids are considered as the four main circulating haemocyte types in Lepidopteran species (4). In particular, granulocytes and plasmatocytes together usually comprise more than 50% of the hemocytes in circulation at the larval stage (3). In our analysis, an effort was made to assign the different hemocyte clusters in scRNAseq to the four major cell types (**Figure 6**). However, these four main hemocyte types become strikingly depleted in the hemolymph of BmNPV-infected silkworms (**Figures 3B, D**). Subsequent verification experiments also confirmed that the prohemocytes accounted for the largest proportion of BmNPV-infected silkworms, and hemocytes in control silkworms corresponded for the largest proportion to granulocytes (**Figure 7**). It can therefore be speculated that the hemocytes of clusters 1, 2, 3, 9, 11, 13, and 18, that appear mainly in the BmNPV-infected silkworm, could correspond to progenitor cells (prohemocytes) that are released from the hematopoietic organs (48) as a response to hemocyte depletion during BmNPV infection.

### Pseudo-Time Trajectories of Silkworm Hemocyte Clusters

**Figure 8:**
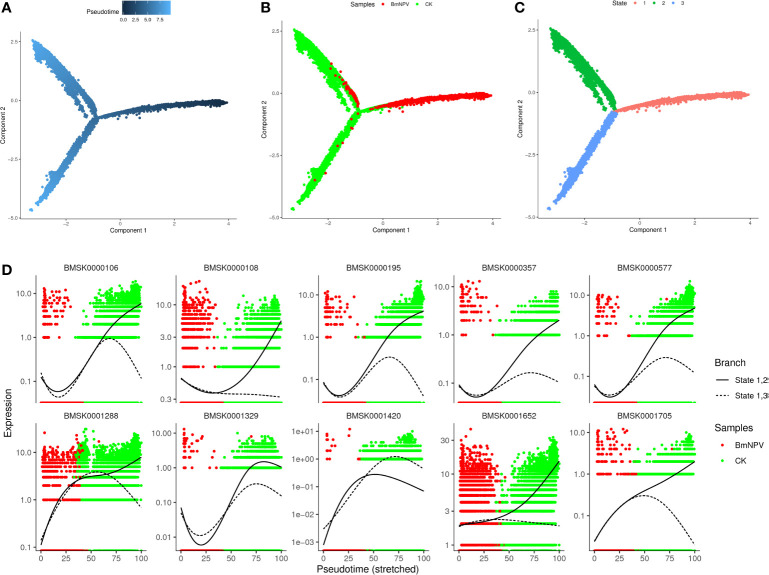
Pseudo-time trajectories of silkworm hemocyte clusters. **(A)** Pseudotime trajectory calculated from all cells in control and BmNPV-infected groups. Prohemocytes are defined as the starting point of the differentiation trajectory. Darker dot colors represent shorter pseudotime and earlier differentiation period. **(B)** Distribution of cells in different samples in cell trajectories. **(C)** Distribution of cells in different states of differentiation in cell trajectories. **(D)** Pseudo-time expression trajectories of branch-dependent top 10 DGEs.

We next sought to investigate the developmental trajectories of different hemocyte clusters in both BmNPV-infected and control samples and for that purpose the pseudo-times were constructed from all single-cell transcriptomics data using Monocle 2 (37). We found that the hemocytes of BmNPV-infected silkworms (the main component is PR) and the hemocytes of normal silkworms (the main components are GR, OE, PL) are obviously in different branches of the pseudo-time differentiation trajectory and present three states of differentiation (**Figures 8A–C**). Prohemocytes are hypothesized to be progenitors that differentiate into one or more of the other hemocyte types (5, 6). Therefore, prohemocytes are defined as the starting point of the differentiation trajectory, and located in state 1 (Cluster 1, 2, 3, 9, 11, 13, 18) (**Figures 8A, C** and **Figure S3**). GR (Cluster 0, 4, 7, 10, 17) and SP (Cluster 19) are mainly located in state 2, and OE (Cluster 5, 12, 16) are predominantly detected in state 3 (**Figure 8C**, **Figure S3**). PL (Cluster 14, 15) is principally found at the intersection of the three differentiation trajectories (**Figure 8C**, **Figure S3**). Accordingly, we speculate that PR (state 1) can first differentiate into PL after which two diverging paths of differentiation will yield GR and SP (state 2) or OE (state 3). While we present here a model for the differentiation pathway of silkworm hemocytes, it should be considered an open issue that needs further experimental verification. **Figure 8D** showed the top 10 genes involved in the differentiation trajectory of silkworm hemocytes. These genes might play important role in the differentiation of silkworm hemocytes.

## Discussion

Hemocytes, the motile cells that populate the hemolymph in insects, can carry out multiple and diverse functions. Similar to macrophages in vertebrates, differentiated hemocytes play an important role in body homeostasis through tissue repair (e.g. production of extracellular matrix around differentiating tissues) and disposal of cellular waste (e.g. phagocytosis of debris from apoptotic cells) (49). In addition, hemocytes play a crucial role in both cellular (e.g. phagocytosis of microbial pathogens, encapsulation of large structures such as eggs from parasitoid eggs) and humoral immunity (e.g. production of AMPs in response to activation of immune signaling pathways) (50). Hemocytes are also involved in the uptake and storage of lipids and nutrients (22).

To understand their diverse functions, a detailed classification of the different types of hemocytes is necessary. With respect to Lepidoptera, morphological and biochemical properties have led to the designation of five major hemocyte cell types (mentioned above) (3, 48) which should be considered only as a very rough approximation for the expected much higher diversity at the molecular level. In *Drosophila*, only three major hemocyte types are distinguished (plasmatocytes, crystal cells, lamellocytes) that were correlated to the five types in Lepidoptera (3, 48). However, compared to Lepidoptera, genetic characterization of hemocyte lineages in *Drosophila* is much more advanced and clear molecular markers for the three different lineages were identified (51, 52). ScRNA-seq analysis of hemocytes confirmed the three lineages in *Drosophila*, although a much higher complexity was revealed at the molecular level. More specifically, the broad categories of plasmatocytes, crystal cells and lamellocytes were found to be distributed into 12, 2 and 2 cell clusters, respectively, that are thought to reflect functional diversification within a broad cell type or represent different stages of maturation (21–23). Attempts were made to classify hemocytes in Lepidoptera at the molecular level, e.g. through the use of specific antibodies, but their molecular targets were never elucidated (53).

In this work, the technique of scRNA-seq was applied to categorize different hemocyte populations in 5^th^ instar larvae of the silkworm that were either mock-infected (injection with PBS) or infected with a high dose of BmNPV for a period of three days. Twenty different hemocyte clusters were identified during joint analysis of the scRNA-seq data from both treatments revealing high heterogeneity among the hemocytes that populate the hemolymph in larvae (of note, when scRNA-seq of the two samples were analyzed separately, highly similar results were obtained, reflecting the robustness of the approach that was used - see **Supplementary Information**). Strikingly, cell clusters that are prevalent in the BmNPV-infected group (1, 2, 3, 9, 11, 13, and 18) were clearly separated from clusters that were specific for the control group (0, 4, 5, 7, 8, 10, 12, 16, 17 and 19). Thus, baculovirus infection clearly has a major impact on the hemocyte population after three days of infection. This may be related to the collection of samples from the late stage of infection. To obtain a more complete picture, samples could be taken from the early stage of infection and subjected to the same type of analysis in the future. Because analysis was carried out at the late stage of infection, it can be speculated that hemocytes of clusters 1, 2, 3, 9, 11, 13, and 18, that appear mainly in the BmNPV-infected silkworms, could correspond to progenitor cells (prohemocytes) that are released from the hematopoietic organs (48) as a response to hemocyte depletion during BmNPV infection.

Our analysis and division of hemocytes in different clusters allowed to assign particular “marker genes” (top 5 of differentially expressed genes) to each cluster (**Figure 1**). However, the number of differentially expressed genes was much higher in uninfected cells than in infected cells, revealing the impact of baculovirus infection. For the uninfected condition, the “marker genes” in the different clusters could give important clues about the functional specialization of the cells that belong to the cluster. However, since there are no recognized marker genes for various types of silkworm hemocytes, we cannot use known marker genes to classify silkworm hemocyte with confidence. Fortunately, some researchers have studied the genes expression in different silkworm hemocytes. Based on the analysis of data in the literature (7, 46, 47), we have tentatively assigned the different clusters to the particular cell types that were described previously during morphological and cytochemical observations (i.e. prohemocytes, plasmatocytes, granulocytes, oenocytoids, spherulocytes) (**Figures 6** and **7**). Additionally, we also verified our classification of BmNPV-infected and control silkworm hemocytes by acridine orange/propidium iodide double-staining, and found it largely consistent with the classification results of scRNA-seq. However, the results of our study cannot yet be regarded as a conclusive classification of silkworm hemocytes. A major contribution of our study may be the identification of potential sets of marker genes that are enriched in hemocyte clusters but require further validation in functional experiments. In future research, the combination of multiple monoclonal antibodies and morphological features could be used to isolate more defined hemocyte populations by flow sorting and achieve a more accurate and comprehensive classification of silkworm hemocyte types.

Baculovirus infections are well known for their virulent character and their large impact on host cellular physiology (54). During baculovirus infection of tissue culture cells, host mRNAs decrease to less than 10% after 48 hr while total (mainly viral) mRNAs increase by 70% (55). In the baculovirus expression vector system, host genes that show an increase in expression relate to the stress response to unfolded proteins indicating that the cellular expression capacity is at its limit (56). However, most studies that monitor the transcriptional response of the host cells to baculovirus infection investigate mainly early responses and are limited to 48 hr post-infection (54), while in this study, scRNA-seq was applied on hemocyte samples after 3 days of infection. Compared to the control sample, a large number of genes are upregulated after BmNPV infection at 3 days in GR (Cluster 0, 6, 10) and OE (Cluster 5) (**Figure 5A**). Among these upregulated genes, 20, 6, and 24 are known silkworm immune-related genes that were significantly induced by BmNPV in clusters 5, 6, and 10, respectively (**File S6**). At the same time, most immune-related genes are inhibited by BmNPV infection, especially in cluster 6 and 10 (**File S6**). However, it should be noted that the number of cells in these clusters of BmNPV-infected silkworms is very small, which greatly limits the interpretation about the possible function of up-regulated immune-related genes. In addition, we noticed that a certain number of genes in PR cell clusters 1 and 2 were also significantly induced by BmNPV, which mainly encode ribosomal proteins (**Figure 5A**, **File S6**). This may be related to the fact that the hypothetical PR cells that are released from the hematopoietic organs have become infected with BmNPV and become reprogrammed to accommodate the synthesis of a large amount of viral protein for virus replication. It is well established that PL and GR are involved in most cellular defense responses (2, 3). These two cell types (PL and GR) were largely decreased after 3 days of BmNPV infection, which will inevitably incapacitate the silkworm’s immune system against BmNPV infection.

Prohemocytes comprise a multipotent precursor cell population that gives rise to other hemocyte subsets (5). Previous research has reported that silkworm larval hemocytes consist of two lineages with the capability of differentiating toward either GR or OE (6). In the present study, pseudo-time trajectories of silkworm hemocyte clusters showed that progression from PR as starting points results in the bifurcation into two branches where OE and GR are located, thus confirming Nakahara et al.’s views on the two lineages of silkworm blood cells (6). The specific differentiation path of each hemocyte type and the function of differentiation-related genes need to be further studied.

**Figure 9:**
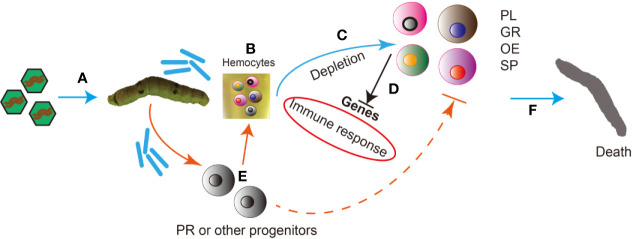
Hypothetical timeline of the natural infection process of silkworms by BmNPV. **(A)** BmNPV occlusion bodies are ingested by the silkworm and become dissolved in the midgut, and occlusion derived virions (ODV) are released which then infect midgut epithelial cells. **(B)** Budded viruses (BV) are produced which spread the infection to the entire silkworm including all hemocytes. **(C)** Viral infection causes severe depletion of the main silkworm hemocyte subgroups including Granulocytes (GR), Plasmatocytes (PL), Oenocytoids (OE) and Spherulocytes (SP). **(D)** The host RNAi and immune responses in silkworm hemocytes were inhibited by BmNPV infection at the late stage. **(E)** In order to replenish the lost hemocytes including GR, PL, OE and SP, the host mobilizes a large number of progenitor cells such as prohemocytes into the circulating hemolymph. However, after the virus infects these progenitor cells, it may prevent them from continuing to differentiate into other types of hemocytes. Therefore, the exhaustion of all blood cells is inevitable. **(F)** Due to the exhaustion of the main hemocyte subsets and the severe suppression of the immune response, the silkworm’s antiviral system collapses causing death.

Based on the results of this research, we present the following hypothetical timeline of the natural infection process of silkworms by BmNPV: first, BmNPV occlusion bodies are ingested by the silkworm and become dissolved in the midgut, and occlusion derived virions (ODV) are released which then infect midgut epithelial cells (**Figure 9A**). Subsequently, budded viruses (BV) are produced which spread the infection to the entire silkworm including all hemocytes (**Figure 9B**). Viral infection causes severe depletion of the main silkworm hemocyte subgroups including GR, PL, OE and SP (**Figure 9C**). Furthermore, the host RNAi and immune responses in silkworm hemocytes were inhibited by BmNPV infection at the late stage (**Figure 9D**). In order to replenish the lost hemocytes including GR, PL, OE and SP, the host mobilizes a large number of progenitor cells such as prohemocytes into the circulating hemolymph. However, after the virus infects these progenitor cells, it may prevent them from continuing to differentiate into other types of hemocytes. Therefore, the exhaustion of all blood cells is inevitable (**Figure 9E**). Due to the exhaustion of the main hemocyte subsets and the severe suppression of the immune response, the silkworm’s antiviral system collapses causing death (**Figure 9F**).

In summary, our scRNA-seq study on hemocytes in the silkworm represents a rich resource of data that can be mined for future experiments to address the function of these cells during development and in response to pathogenic infection in Lepidoptera. The “marker genes” that were assigned to different clusters need to be confirmed by independent methods such as *in situ* hybridization and immunocytochemistry with specific antibodies. With respect to the control group, experimental manipulation (injection and wounding) and developmental stage (following the molt) could have made an impact on the composition of hemocytes in the analysis of the scRNAseq data (i.e. biased towards tissue remodeling). Checking hemocyte populations at different developmental stages or after particular experimental interventions (e.g. RNAi, hormones, cytokines) will lead to additional insights. Regarding BmNPV infection, earlier time points or lower doses of virus may provide new insights into the vulnerability of particular hemocyte types and the mounting of an immune response. Our study confirms that the technique of scRNA-seq provides a great advancement for the study of hemocytes as well as other tissue types and will lead to a more complete understanding of biological processes and organ function.

## Data Availability Statement

The datasets presented in this study can be found in online repositories. The names of the repository/repositories and accession number(s) can be found below: https://www.ncbi.nlm.nih.gov/, PRJNA658439.

## Author Contributions

MF and LS participated in the design of the study, collected and analyzed data and drafted the manuscript. JX, SF, RP, XW, YZ, and PW helped with sample preparation and data analysis. JS participated in the design and coordination of the study, and revised the manuscript. All authors contributed to the article and approved the submitted version.

## Funding

This work was supported by the National Natural Science Foundation of China (31872426), Guangdong Natural Science Foundation (2018A030310210); Guangdong Provincial Promotion Project on Preservation and Utilization of Local Breed of Livestock and Poultry (No.2018-143); and South China Agricultural University high-level talent launch project.

## Conflict of Interest

The authors declare that the research was conducted in the absence of any commercial or financial relationships that could be construed as a potential conflict of interest.

## Supplementary Material

Supplementary Figure 1 Separate analysis of hemocytes of the groups of control and BmNPV infection. **(A, B)** The hemocytes of the separately analyzed groups of BmNPV infection **(A)** and control **(B)** were compared with the joined analyzed set (BmNPV+CK) using cell-specific barcodes. The agreement between the two clustering approaches is relatively high, especially for the cells in the control group **(B)**. **(C)** The hypervariable genes that were identified for cell cluster classification in scRNA-seq were compared between individually analyzed groups and the unified set.

Supplementary Figure 2 Volcano plot of identified DEGs between BmNPV-infected and control groups in clusters 0 **(A)**, 1 **(B)**, 2 **(C)**, 3 **(D)**, 4 **(E)**, 5 **(F)**, 6 **(G)**, 7 **(H)**, 8 **(I)**, 10 **(J)**, 11 **(K)**, 14 **(L)**, 15 **(M)** and 19 **(N)**.

Supplementary Figure 3 Distribution of each silkworm hemocyte cluster on pseudotime trajectories.

Supplementary File 1 scRNA-seq data statistics.

Supplementary File 2 Up-regulated differentially expressed genes (DEGs) in each silkworm hemocyte cluster.

Supplementary File 3 Top 5 DEGs of each silkworm hemocyte cluster.

Supplementary File 4 Immune-related DEGs selected from clusters 0 to 19 and their distribution in each cluster.

Supplementary file 5 Statistics of virus-infected cells and expression of viral genes in the different clusters of the BmNPV-infected group.

Supplementary File 6 Properties of identified DEGs in clusters 0, 1, 2, 3, 4, 5, 6, 7, 8, 10, 11, 14, 15 and 19 between the BmNPV-infected and control groups.
