# Supplementary figures and images for: Identification of Silkworm Hemocyte Subsets and Analysis of Their Response to Baculovirus Infection Based on Single-Cell RNA Sequencing
Source: Front Immunol. 2021 Apr 30;12:645359. doi: 10.3389/fimmu.2021.645359 (PMC8119652; doi:10.3389/fimmu.2021.645359)

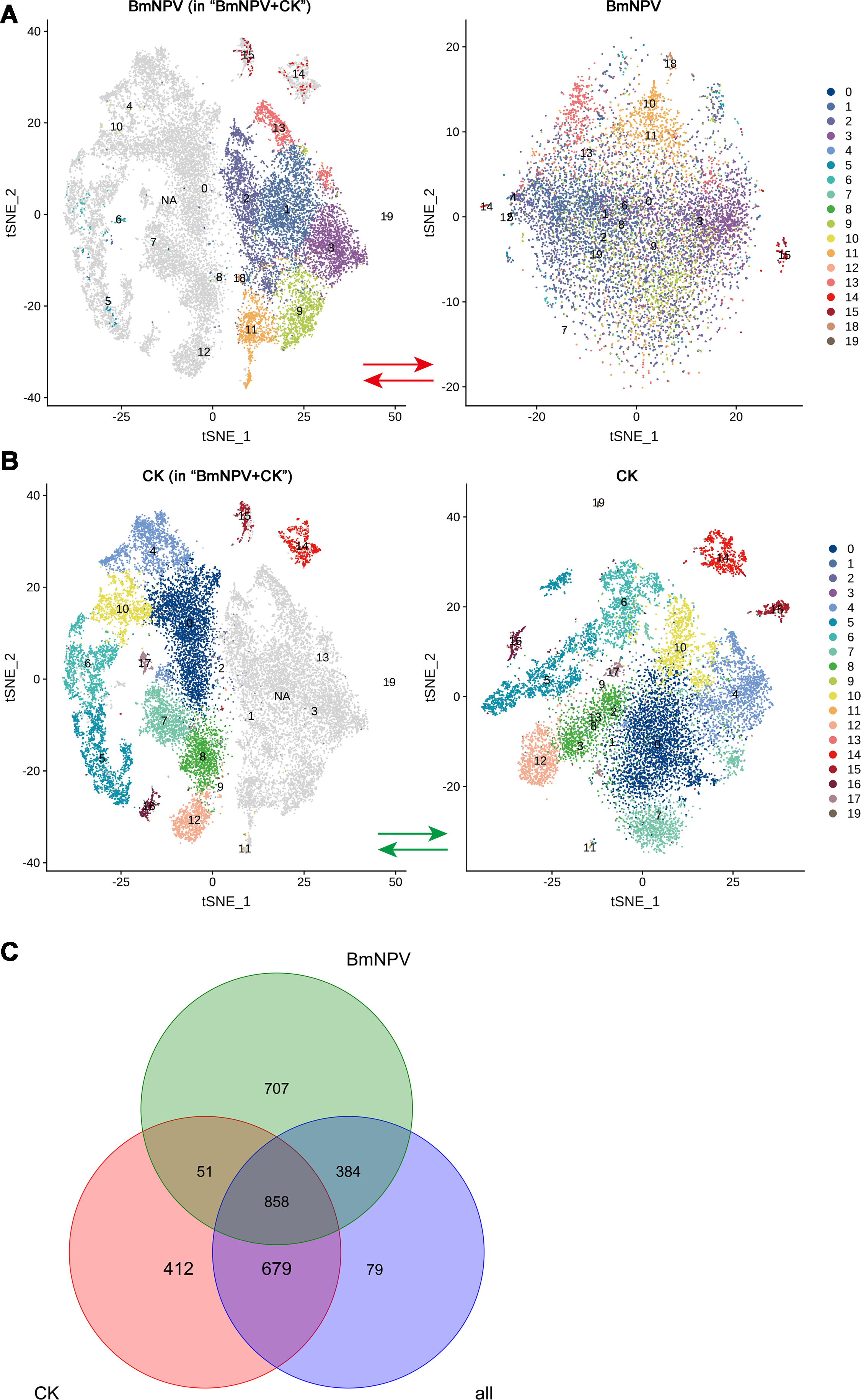

Supplement: Supplementary Figure 1 — Separate analysis of hemocytes of the groups of control and BmNPV infection. (A, B) The hemocytes of the separately analyzed groups of BmNPV infection (A) and control (B) were compared with the joined analyzed set (BmNPV+CK) using cell-specific barcodes. The agreement between the two clustering approaches is relatively high, especially for the cells in the control group (B). (C) The hypervariable genes that were identified for cell cluster classification in scRNA-seq were compared between individually analyzed groups and the unified set. [file Image_1.tif]

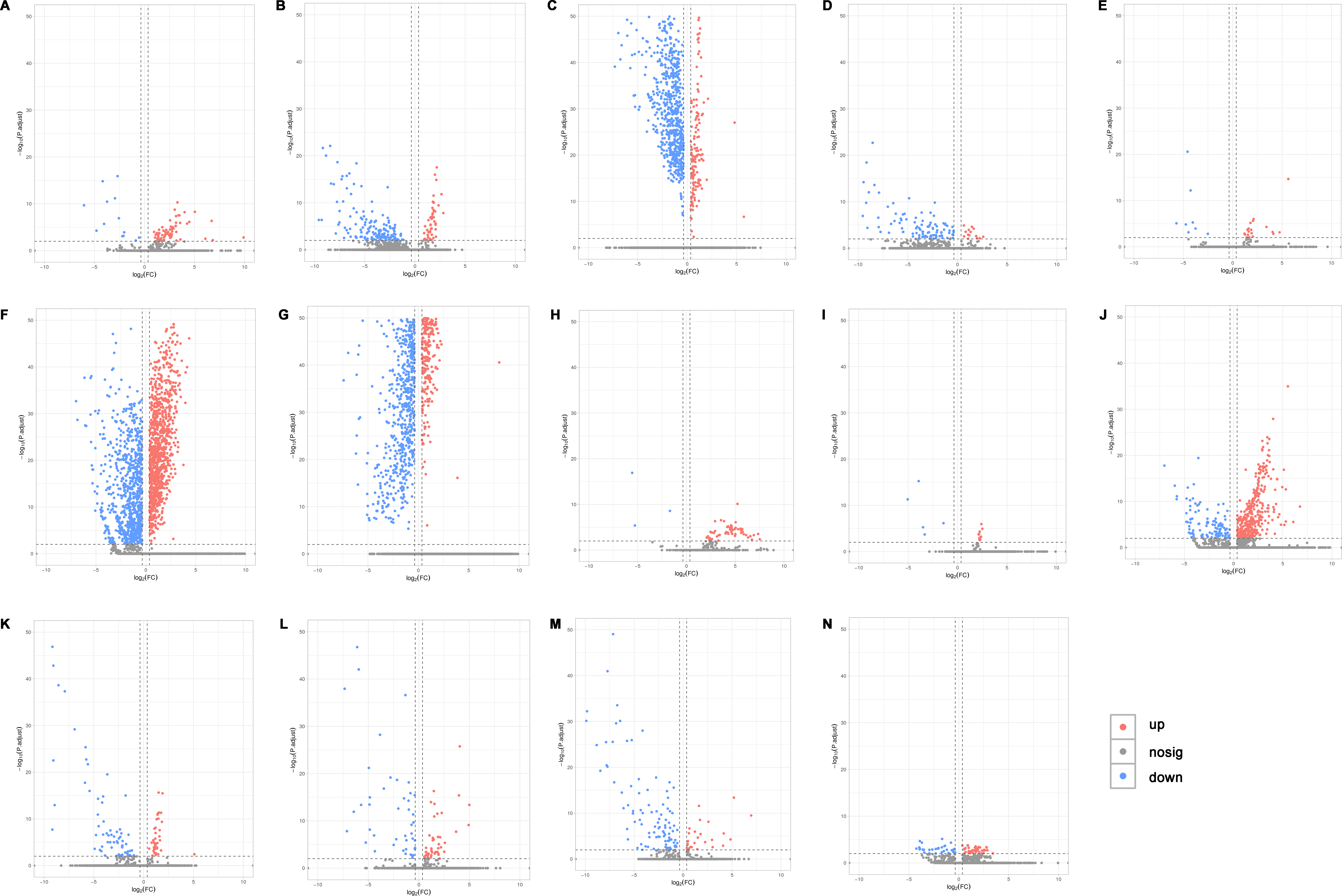

Supplement: Supplementary Figure 2 — Volcano plot of identified DEGs between BmNPV-infected and control groups in clusters 0 (A), 1 (B), 2 (C), 3 (D), 4 (E), 5 (F), 6 (G), 7 (H), 8 (I), 10 (J), 11 (K), 14 (L), 15 (M) and 19 (N). [file Image_2.tif]

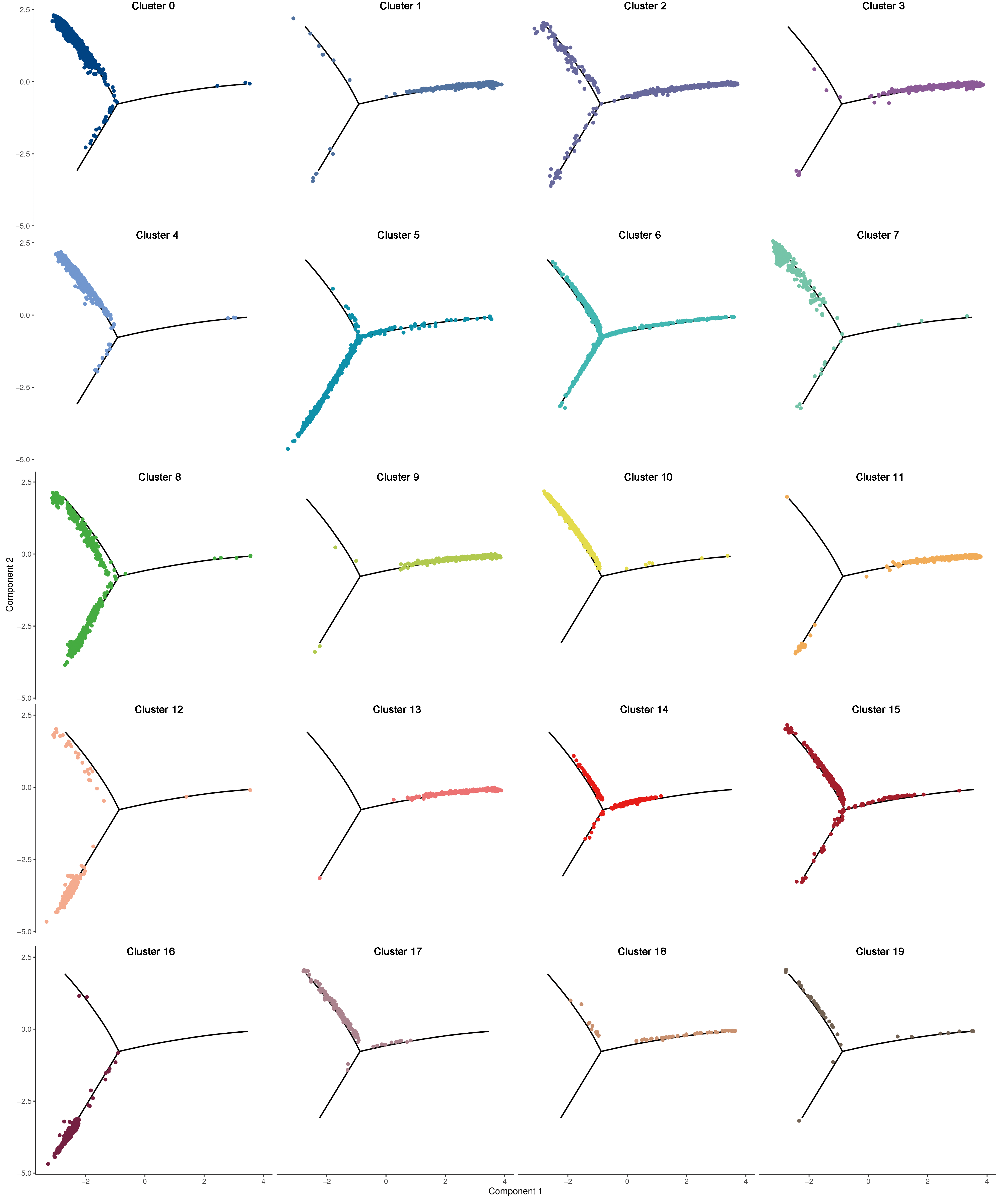

Supplement: Supplementary Figure 3 — Distribution of each silkworm hemocyte cluster on pseudotime trajectories. [file Image_3.tif]
